# Draft genome sequence of *Arthrobacter* sp. strain B6 isolated from the high-arsenic sediments in Datong Basin, China

**DOI:** 10.1186/s40793-017-0231-9

**Published:** 2017-01-23

**Authors:** Linghua Xu, Wanxia Shi, Xian-Chun Zeng, Ye Yang, Lingli Zhou, Yao Mu, Yichen Liu

**Affiliations:** 1 State Key Laboratory of Biogeology and Environmental Geology & Department of Biological Science and Technology, School of Environmental Studies, China University of Geosciences (Wuhan), Wuhan, China; 2 School of Chemistry and Chemical Engineering, Hubei Polytechnic University, Huangshi, China

**Keywords:** *Arthrobacter* sp. B6, Genome, Arsenate reduction, High-arsenic sediment, Datong basin

## Abstract

*Arthrobacter* sp. B6 is a Gram-positive, non-motile, facultative aerobic bacterium, isolated from the arsenic-contaminated aquifer sediment in the Datong basin, China. This strain displays high resistance to arsenic, and can dynamically transform arsenic under aerobic condition. Here, we described the high quality draft genome sequence, annotations and the features of *Arthrobacter* sp. B6. The G + C content of the genome is 64.67%. This strain has a genome size of 4,663,437 bp; the genome is arranged in 8 scaffolds that contain 25 contigs. From the sequences, 3956 protein-coding genes, 264 pseudo genes and 89 tRNA/rRNA-encoding genes were identified. The genome analysis of this strain helps to better understand the mechanism by which the microbe efficiently tolerates arsenic in the arsenic-contaminated environment.

## Introduction

The genus *Arthrobacter* was first proposed in 1947 by Conn and Dimmick [1], belongs to the family of *Micrococcaceae* in the class of *Actinobacteria* *.* Recently, based on the intrageneric phylogeny and chemotaxonomic characteristics, the description of the genus *Arthrobacter* *sensu lato* was emended by Busse, and the genus *Arthrobacter* *sensu stricto* was restricted to *A. globiformis*, *A. pascens*, *A. oryzae* and *A. humicola* [2]. Due to their nutritional versatility and tolerance to various environmental stressors [3–7], *Arthrobacter* species are widely present in soils and the environments contaminated with chemicals and heavy metal [8–13], as well as extreme environments, such as Antarctic and radioactive sediments [14, 15].

*Arthrobacter* sp. B6 was isolated from an arsenic-contaminated sediment sample collected from the Datong Basin, China, where the uses of high arsenic groundwater for drinking and irrigation have resulted in endemic arsenic poisoning among tens of thousands of residents [16]. Strain B6 is of particular interest because it showed high level of resistance to arsenic and can dynamically transform arsenic under aerobic condition. Here, we presented a summary of the taxonomic characterization of *Arthrobacter* sp. B6 and its main genomic features. These data help to better understand the microbial detoxification mechanism for arsenic, and are useful for the comparisons of the genomic and physiological features between this isolate and other *Arthrobacter* species.

## Organism information

### Classification and features

**Fig. 1:**
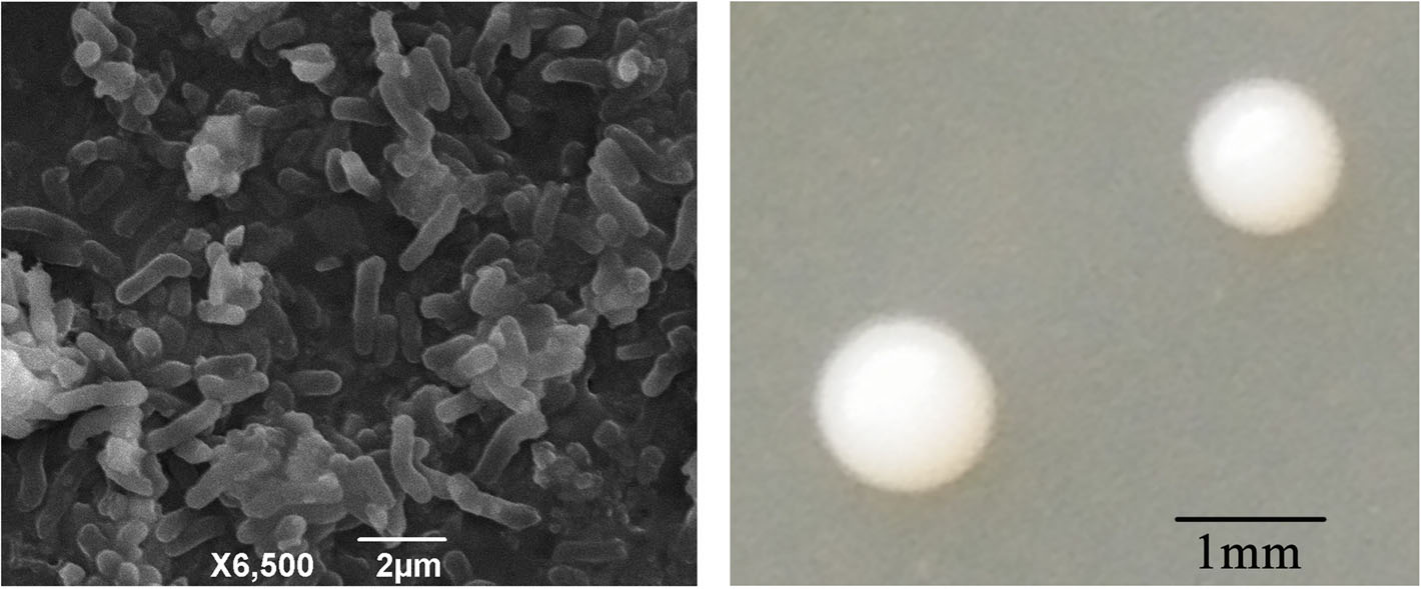
Images of *Arthrobacter* sp. B6 using scanning electron microscopy (Left) and the appearance of colony morphology on 0.1× Trypticase Soy Broth solid media (Right)

*Arthrobacter* sp. B6 is a Gram-positive, non-motile, facultative aerobic bacterium. Cells are straight or slightly curved rods during log phase of bacterial growth (Fig. 1) and become coccoid in stationary phase. The bacteria cells formed white colonies on 0.1× Trypticase Soy Broth agar plate. Colonies are convex and circular with entire margin. The strain can grow at a wide range of temperatures from 4 to 37 °C; the optimum is 30 °C. It can proliferate in a pH range of 6.0–8.5; the optimum is 7.0. The strain tolerates high concentrations of NaCl up to approximately 7% (Table 1). It is catalase- and oxidase-positive. It hydrolyzes starch and tyrosine, but not o-nitrophenyl-β-d-galactoside, gelatin, aesculin, chitin, casein or cellulose. It is negative for nitrate reduction, H_2_S production, citrate utilization, indole production, arginine dihydrolase and urease activity.

**Table 1:** Classification and general features of *Arthrobacter* sp. B6 [19]

MIGS ID	Property	Term	Evidence code^a^
	Classification	Domain *Bacteria*	TAS [24]
		Phylum *Actinobacteria*	TAS [25]
		Class *Actinobacteria*	TAS [26]
		Order *Actinomycetales*	TAS [27, 28]
		Family *Micrococcaceae*	TAS [27, 29]
		Genus *Arthrobacter*	TAS [1, 2]
		Species undetermined	-
		Strain: B6	IDA
	Gram stain	Positive	IDA
	Cell shape	Polymorphic: rod to coccus shaped	IDA
	Motility	Non-motile	IDA
	Sporulation	Non-sporulating	IDA
	Temperature range	4–37 °C	IDA
	Optimum temperature	30 °C	IDA
	pH range; Optimum	6.0–8.5; 7	IDA
	Carbon source	Dextrin, Tween 40, D-fructose, Gentiobiose, α-D-glucose, Lactulose, Maltotriose, D-mannose, D-mannitol, D-melezitose, Palatinose, D-psicose, D-raffinose, L-rhamnose, D-ribose, D-sorbitol, Sucrose, Turanose, α- hydroxybutyric acid, α-ketoglutaric acid, L-malic acid, Pyruvic acid, D-alanine, L-alanine, L-serine, Glycerol, Adenosine, 2-deoxy adenosine, Inosine.	IDA
MIGS-6	Habitat	Soil, sediment	IDA
MIGS-6.3	Salinity	1–7% NaCl (w/v)	IDA
MIGS-22	Oxygen requirement	Aerobic	IDA
MIGS-15	Biotic relationship	free-living	IDA
MIGS-14	Pathogenicity	Non-pathogen	NAS
MIGS-4	Geographic location	Datong basin, Shanxi, China	IDA
MIGS-5	Sample collection	August 2011	IDA
MIGS-4.1	Latitude	39.4899	IDA
MIGS-4.2	Longitude	112.915	IDA
MIGS-4.4	Altitude	Not recorded	

^a^Evidence codes - IDA: Inferred from Direct Assay; TAS: Traceable Author Statement (i.e., a direct report exists in the literature); NAS: Non-traceable Author Statement (i.e., not directly observed for the living, isolated sample, but based on a generally accepted property for the species, or anecdotal evidence). These evidence codes are from the Gene Ontology project [30]

The strain utilizes dextrin, tween 40, D-fructose, gentiobiose, α-D-glucose, lactulose, maltotriose, D-mannose, D-mannitol, D-melezitose, palatinose, D-psicose, D-raffinose, L-rhamnose, D-ribose, D-sorbitol, sucrose, turanose, α- hydroxybutyric acid, α-ketoglutaric acid, L-malic acid, pyruvic acid, D-alanine, L-alanine, L-serine, glycerol, adenosine, 2-deoxy adenosine and inosine as tested using the Biolog GP2 microplate system. The major fatty acids of strain B6 are anteiso-C15:0 (56.58%), anteiso-C17:1ω9c (8.89%), anteiso-C17:0 (8.22%), iso-C15:0 (7.63%), iso-C16:0 (5.26%), sum in feature 3 (4.31%), summed feature 3 (containing C16:1ω6c and/or C16:1ω7c) (4.31%) and iso-C16:1 H (2.32%). These data suggested that the morphological and biochemical traits and fatty acid profile of B6 are consistent with those of other described species of the genus *Arthrobacter*.

**Fig. 2:**
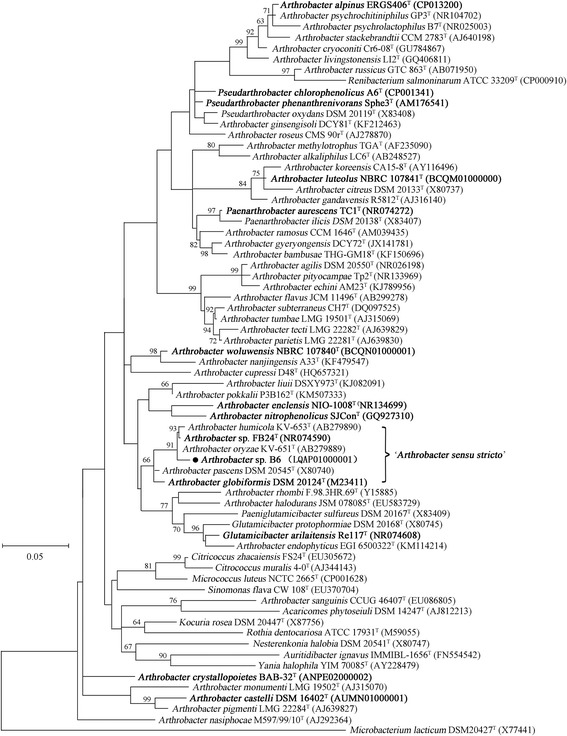
Phylogenetic tree based on 16S rRNA gene sequences showing the phylogenetic position of *Arthrobacter* sp. B6 (●). Sequences were aligned with the CLUSTAL W program and were constructed using maximum-likelihood method implemented in MEGA 6.0 program [17, 18]. GenBank accession numbers are listed in parentheses. Type strains are indicated with a superscript T. Strains with published genomes are shown in bold. Bootstrap support values for 1000 replications above 50% are shown near nodes. The scale bar indicates 0.05 nucleotide substitution per nucleotide position

The 16S rRNA gene sequence of strain B6 shares 94.67–99.59% identities with those of other known species of the genus *Arthrobacter*. In order to evaluate the evolutionary relationships between B6 and other known strains of the genus *Arthrobacter*, the 16S rRNA gene sequence of all of these bacteria were aligned using ClustalW [17], and a phylogenetic tree were conducted using the maximum-likelihood and neighbor-joining algorithms implemented in MEGA 6.0, respectively [18]. The phylogeny illustrated that the strain B6 is closely associated with *Arthrobacter oryzae*, *A. globiformis*, *A. pascens* and *A. humicola*; suggesting that B6 is affiliated with the genus *Arthrobacter* (Fig. 2). We also found that *Arthrobacter* sp. B6 showed high resistance to arsenic, with maximal inhibitory concentrations of 150.0 mM for arsenate and 5.0 mM for arsenite. A dynamic transformation of arsenic catalyzed by strain B6 was observed when it was cultured aerobically with arsenate.

## Genome sequencing information

### Genome project history

*Arthrobacter* sp. strain B6 was selected for sequencing on the basis of its high resistance to arsenic and dynamic arsenic transformation capability. The Whole Genome Shotgun project has been deposited at DDBJ/EMBL/GenBank database under the accession number LQAP00000000. A summary of the main project information on compliance with MIGS version 2.0 is shown in Table 2 [19].

**Table 2:** Project information

MIGS ID	Property	Term
MIGS 31	Finishing quality	High-Quality Permanent Draft
MIGS-28	Libraries used	Illumina Std. shotgun library
MIGS 29	Sequencing platforms	Illumina HiSeq 2000
MIGS 31.2	Fold coverage	161 ×
MIGS 30	Assemblers	SOAPdenovo v2.04
MIGS 32	Gene calling method	Glimmer v3.02
	Locus Tag	AU175
	Genbank ID	LQAP01000000
	GenBank Date of Release	Jun 15, 2016
	GOLD ID	Gs0118476
	BIOPROJECT	PRJNA306410
MIGS 13	Source Material Identifier	CGMCC 1.15656
	Project relevance	Biotechnological, Environmental

### Growth conditions and genomic DNA preparation

Strain B6 was grown at 30 °C in 0.1× Trypticase Soy Broth liquid medium to mid-exponential phase. Genomic DNA was extracted from 0.5 to 1.0 g of cells using the modified method of Marmur [20]. The purity of DNA, expressed as the value of A260/A280, was assessed on a NanoDrop™ ND-1000 Spectrophotometer (Biolab).

### Genome sequencing and assembly

The draft genome of *Arthrobacter* sp. B6 was sequenced at the Beijing Genomics Institute (BGI, Shenzhen) using the high throughout sequencing technique. A standard Illumina shotgun library was constructed and sequenced using the Illumina HiSeq 2000 platform; this generated 8,355,450 clean reads totaling 752 Mbp. These reads were assembled using the Short Oligonucleotide Analysis Package (SOAPdenovo v2.04) with all parameters set to default [21]. The final draft assembly contains 25 contigs in 8 scaffolds. Final assembly was based on all clean reads that provide an average of 161-fold coverage of the genome. The total size of the genome is 4.66 Mbp.

### Genome annotation

Genes were identified using Glimmer v3.02 [22]. The predicted CDSs were translated into amino acid sequences that were used as queries to BLAST the GenBank, Swissprot, InterPro, KEGG, COG and GO databases, respectively. These data were combined to assert a product description for each predicted protein. Additional gene prediction analysis and functional annotation was performed using the Integrated Microbial Genomes-Expert Review (IMG-ER) platform [23].

## Genome properties

**Fig. 3:**
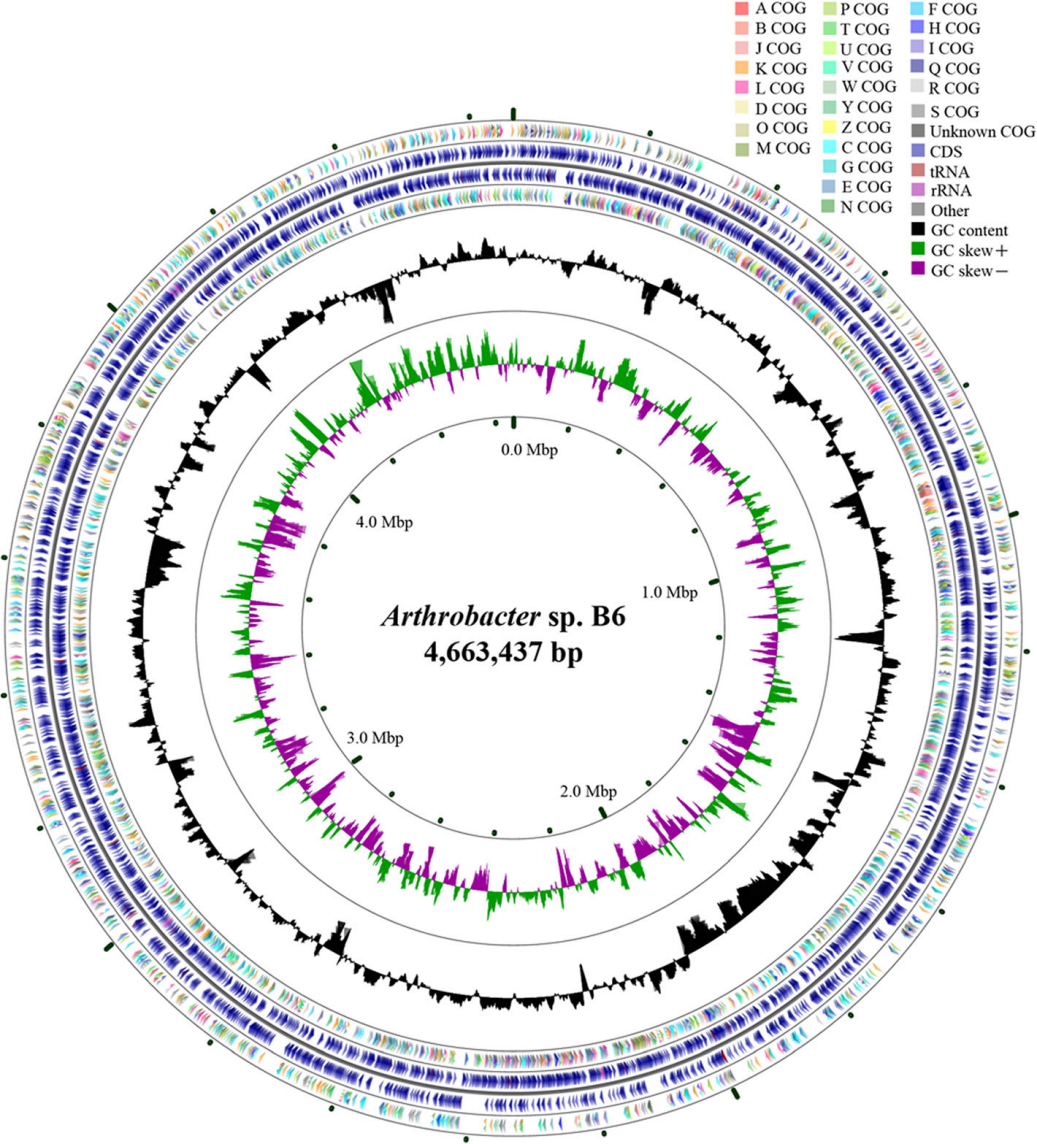
A graphical circular map of the genome performed with CGview comparison tool [31]. From outside to center, ring 1 and 4 show protein-coding genes oriented in the forward (colored by COG categories) and reverse (colored by COG categories) directions, respectively. ring 2 and 3 denote genes on forward/reverse strand; ring 5 shows G + C% content plot, and the inner-most ring shows GC skew, purple indicating negative values and olive, positive values

The assembly of the draft genome sequence consists of 8 scaffolds amounting to 4,663,437 bp. The G + C content is 64.67% (Table 3). From the genome, 4309 genes were predicted, of which 3956 are protein-coding genes. Among these protein-coding genes, 154 were assigned to putative functions, and 275 were annotated as hypothetical proteins. The assignment of genes into COGs functional categories is presented in Table 4 and Fig. 3.

**Table 3:** Genome statistics

Attribute	Value	% of Total
Genome size (bp)	4,663,437	100.00
DNA coding (bp)	4,100,739	87.93
DNA G + C (bp)	3,015,845	64.67
DNA scaffolds	8	100.00
Total genes	4309	100.00
Protein coding genes	3956	91.81
RNA genes	89	2.07
Pseudo genes	264	6.12
Genes in internal clusters	4250	98.63
Genes with function prediction	3527	81.85
Genes assigned to COGs	2210	51.29
Genes with Pfam domains	3464	80.39
Genes with signal peptides	220	5.11
Genes with transmembrane helices	249	5.78
CRISPR repeats	125	2.90

**Table 4:** Number of genes associated with general COG functional categories

Code	Value	%age	Description
J	145	6.56	Translation, ribosomal structure and biogenesis
A	1	0.05	RNA processing and modification
K	162	7.33	Transcription
L	110	4.98	Replication, recombination and repair
B	1	0.05	Chromatin structure and dynamics
D	12	0.54	Cell cycle control, Cell division, chromosome partitioning
V	26	1.18	Defense mechanisms
T	58	2.62	Signal transduction mechanisms
M	72	3.26	Cell wall/membrane biogenesis
N	0	0	Cell motility
U	18	0.81	Intracellular trafficking and secretion
O	65	2.94	Posttranslational modification, protein turnover, chaperones
C	168	7.60	Energy production and conversion
G	225	10.18	Carbohydrate transport and metabolism
E	272	12.31	Amino acid transport and metabolism
F	71	3.21	Nucleotide transport and metabolism
H	111	5.02	Coenzyme transport and metabolism
I	103	4.66	Lipid transport and metabolism
P	127	5.75	Inorganic ion transport and metabolism
Q	66	2.99	Secondary metabolites biosynthesis, transport and catabolism
R	266	12.04	General function prediction only
S	131	5.93	Function unknown
-	2099	48.71	Not in COGs

The total is based on the total number of protein coding genes in the genome

## Insights from the genome sequence

Genome comparison using the RAST Prokaryotic Genome Annotation Server revealed that the genome sequence of *Arthrobacter* sp. B6 is most similar to that of *Arthrobacter* sp. FB24 (comparison score: 536), but less similar to those of other *Arthrobacter* strains. *Arthrobacter* sp. B6 shares 2035, 2011, 1958, 1930, 1850 and 1829 genes with the strains *A. globiformis* NBRC 12137, *Arthrobacter* sp. FB24, *A. enclensis* NIO-1008, *A. nitrophenolicus* SJCon, *A. castelli* DSM 16402 and *A. crystallopoietes* BAB-32, respectively.

A three-gene (*arsR-acr*3-*arsC*) operon involved in the regulation of arsenate tolerance and reduction was identified from the genome of *Arthrobacter* sp. B6. The putative arsenate reductase (ArsC) of strain B6 shows 96% and 95% sequence identities to those of *Arthrobacter* sp. Leaf137 and *Pseudarthrobacter phenanthrenivorans* Sphe3, respectively. It also shows 89% identities to those of *A. globiformis* NBRC 12137, *A. nitrophenolicus* SJCon, *A. enclensis* NIO-1008 and *Arthrobacter* sp. FB24, respectively. The amino acid sequence of ACR3 displays 85% identity to that of the arsenic transporter from *Arthrobacter* sp. FB24. Numerous genes responsible for tolerance or detoxification of metals were identified from the genome of *Arthrobacter* sp. B6, including copper resistance protein CopC and CopD, copper chaperone, copper-translocating P-type ATPase, cobalt-zinc-cadmium resistance protein CzcD, mercuric reductase, DNA gyrase subunit A and B involved in fluoroquinolones resistance, various polyols ABC transporter and DedA protein involved in the uptake of selenate and selenite. In addition, there are some genes in the genome responsible for osmotic stress. The high tolerance of salt (7% NaCl) of strain B6 may be explained by the presence of glycine betaine ABC transport system permease protein in the genome.

## Conclusions

In the present study, we characterized the genome of *Arthrobacter* sp. B6 that was isolated from the arsenic-contaminated aquifer sediment in the Datong Basin, China. It contains numerous genes involved in heavy metal tolerance and detoxification. The knowledge of the genome sequence of *Arthrobacter* sp. B6 lays foundation for better understanding of the special metabolic abilities of the strain and for elucidation of the metabolic diversity of bacteria inhabiting in the high-arsenic environment. Further functional analyses of the identified genes may gain insights into the detailed molecular mechanisms by which the microbes tolerate and transform arsenic in the arsenic-contaminated environments.

## Abbreviations

ABC: ATP-binding cassette
ACR3: Arsenite transporter
ArsC: Arsenate reductase
ArsR: Arsenite responsive repressor
BLAST: Basic local alignment search tool
CDS: Coding DNA sequence
CRISPR: Clustered regularly interspaced short
DedA: Integral membrane protein
IMG-ER: Integrated Microbial Genomes-Expert Review
MIGS: Minimum information on the genome sequence
